# Effectiveness and cost-effectiveness of a physical activity loyalty scheme for behaviour change maintenance: a cluster randomised controlled trial

**DOI:** 10.1186/s12889-016-3244-1

**Published:** 2016-07-22

**Authors:** Ruth F. Hunter, Sarah F. Brennan, Jianjun Tang, Oliver J. Smith, Jennifer Murray, Mark A. Tully, Chris Patterson, Alberto Longo, George Hutchinson, Lindsay Prior, David P. French, Jean Adams, Emma McIntosh, Frank Kee

**Affiliations:** UKCRC Centre of Excellence for Public Health Research (NI)/Centre for Public Health, Institute of Clinical Sciences B, Royal Victoria Hospital, Queen’s University Belfast, Grosvenor Road, Belfast, BT12 6BJ Northern Ireland; UKCRC Centre of Excellence for Public Health Research (NI)/Centre for Public Health, Queen’s University Belfast, Belfast, Northern Ireland; School of Biological Sciences/UKCRC Centre of Excellence for Public Health Research (NI), Queen’s University Belfast, Belfast, Northern Ireland; School of Sociology, Social Policy and Social Work/UKCRC Centre of Excellence for Public Health Research (NI), Queen’s University Belfast, Belfast, Northern Ireland; School of Psychological Sciences, University of Manchester, Manchester, England; Centre for Diet and Activity Research (CEDAR), MRC Epidemiology Unit, University of Cambridge, Cambridge, England; Health Economics and Health Technology Assessment, University of Glasgow, Glasgow, Scotland

**Keywords:** Physical activity, Workplace, Intervention, Cluster RCT, Behaviour change maintenance, Financial incentives, Economic evaluation, Behavioural economics, Mediation analyses

## Abstract

**Background:**

Increasing physical activity in the workplace can provide employee physical and mental health benefits, and employer economic benefits through reduced absenteeism and increased productivity. The workplace is an opportune setting to encourage habitual activity. However, there is limited evidence on effective behaviour change interventions that lead to maintained physical activity. This study aims to address this gap and help build the necessary evidence base for effective, and cost-effective, workplace interventions.

**Methods/design:**

This cluster randomised control trial will recruit 776 office-based employees from public sector organisations in Belfast and Lisburn city centres, Northern Ireland. Participants will be randomly allocated by cluster to either the Intervention Group or Control Group (waiting list control). The 6-month intervention consists of rewards (retail vouchers, based on similar principles to high street loyalty cards), feedback and other evidence-based behaviour change techniques. Sensors situated in the vicinity of participating workplaces will promote and monitor minutes of physical activity undertaken by participants. Both groups will complete all outcome measures. The primary outcome is steps per day recorded using a pedometer (Yamax Digiwalker CW-701) for 7 consecutive days at baseline, 6, 12 and 18 months. Secondary outcomes include health, mental wellbeing, quality of life, work absenteeism and presenteeism, and use of healthcare resources. Process measures will assess intervention “dose”, website usage, and intervention fidelity. An economic evaluation will be conducted from the National Health Service, employer and retailer perspective using both a cost-utility and cost-effectiveness framework. The inclusion of a discrete choice experiment will further generate values for a cost-benefit analysis. Participant focus groups will explore who the intervention worked for and why, and interviews with retailers will elucidate their views on the sustainability of a public health focused loyalty card scheme.

**Discussion:**

The study is designed to maximise the potential for roll-out in similar settings, by engaging the public sector and business community in designing and delivering the intervention. We have developed a sustainable business model using a ‘points’ based loyalty platform, whereby local businesses ‘sponsor’ the incentive (retail vouchers) in return for increased footfall to their business.

**Trial registration:**

ISRCTN17975376 (Registered 19/09/2014).

**Electronic supplementary material:**

The online version of this article (doi:10.1186/s12889-016-3244-1) contains supplementary material, which is available to authorized users.

## Background

The current global physical inactivity ‘pandemic’ [1] requires a determined, innovative response with a particular emphasis on behaviour change maintenance in light of the modest, short term results of interventions to date [2–4]. Interventions in the workplace have the potential to contribute significantly to the formation of behaviour change maintenance [5]. However, there has been an expansion of sedentary occupations, with less than 1 % of adults in Northern Ireland doing at least 10 min of physical activity at work per week [6]. As well as benefitting the physical and mental health of employees, workplace physical activity interventions have the potential to benefit the organisation, through reduced absenteeism and increased productivity; and the wider economy as a whole by keeping people economically active for longer [7]. If such improvements to health are realised, they could lead to lower healthcare utilisation and costs [8]. The costs of lost productivity are estimated to be £5.5 billion a year from sickness absence and £1 billion a year from the premature death of people of working age [7].

Indeed, recent NICE guidelines recommend the promotion of physical activity in and around the workplace, particularly through walking and cycling [4]. However, current evidence to support the effectiveness of such interventions is mixed [9], with reviews [10–12] calling for more robust research on workplace interventions and well-designed RCTs. Previous meta-analyses of workplace physical activity interventions have shown small, positive, short term effects [10–12] on levels of walking but little long term effectiveness is evident [4]. Thus there is a recognised need to develop workplace interventions purposefully designed to encourage physical activity behaviour change maintenance.

The UK and US Governments [13, 14] have encouraged the use of incentives for promoting healthy lifestyles. The use of financial incentives for health-related behaviour change has been met with some scepticism [15], although their acceptability may be contingent on various factors, such as their effectiveness, type, and the target behaviour [16]. There is evidence to support the use of financial incentives for the initiation of behaviour change, for example, smoking, substance abuse [17–20]. Further, recent research has demonstrated evidence of financial incentives for single, one-off heath behaviours, such as vaccinations [19], and encouragingly, habitual health-related behaviour change [20]. However, the evidence for their effect on other health behaviours is sparse. Some financial [21] and non-financial incentives [22] have been shown to increase levels of physical activity, at least in the short-term and mainly with respect to structured exercise programmes [22], rather than free-living physical activity. There is a wealth of evidence on reinforcement of behaviour that shows that whilst incentives may be effective at changing behaviour, the effects are unlikely to be maintained when the incentives are withdrawn in the absence of other interventions [23]. Therefore, financial incentives alone may not be sufficient to promote maintained physical activity behaviour change, but may do so when embedded within an evidence-based, theoretically-driven intervention [16]. Further, financial incentive interventions designed using behavioural economic concepts have been shown to be effective for changing behaviour [24]. There is also limited evidence around the cost-effectiveness of interventions to promote physical activity in the workplace [25, 26], or indeed the cost-effectiveness of financial incentive-based interventions [19]. Therefore, to address such gaps in the evidence base and following the successful completion of a pilot study [27], we aim to conduct a cluster RCT of a complex intervention incorporating financial incentives to encourage physical activity and maintained behaviour change.

## Research objectives

The cluster RCT has the following objectives:

1. To investigate the effectiveness of the intervention to increase employee’s physical activity levels;
2. To investigate if any change in physical activity behaviour is maintained over time;
3. To conduct cost-effectiveness, cost-utility and cost-benefit analyses of the intervention;
4. To investigate how the intervention impacts on other health behaviours and outcomes;
5. To investigate wider work-related effects including sickness absenteeism and work presenteeism;
6. To investigate the mediators of (a) uptake and use of the loyalty card, (b) initiation, and (c) maintenance of behaviour change;
7. To conduct a parallel qualitative study to further identify those who benefitted from the intervention, how and why it worked for them, and explore mediators of behaviour change;
8. To conduct a Discrete Choice Experiment (DCE) to investigate the possible effective levels of incentives for such interventions;
9. To conduct a behavioural economic field experiment on inter-temporal and risk preferences to investigate the relationship between behavioural change, discounting and financial incentives.

## Methods/design

### Design

A workplace-based cluster RCT will evaluate the intervention, incorporating nested behavioural economic experiments, and process evaluation. The study protocol is developed using the MRC framework for complex interventions [28] and SPIRIT guidelines [29] and was successfully tested in a pilot study [27].

### Study population

#### Recruitment of workplaces

The study will target public sector employees involved in predominantly office-based occupations whose workplace is within Belfast or Lisburn City Centres, Northern Ireland. People in predominantly office-based jobs spend a large proportion of their day physically inactive while public sector organisations have been reported to have higher sickness absence rates than private sector workplaces [7, 30, 31]. Public sector organisations will be purposively sampled within 2 km radius of the city centre or offer similar physical activity opportunities within a 2 km radius of their location, have a minimum of 100 employees in predominantly office-based occupations, and have similar opportunities for physical activity in the vicinity of the workplace. Meetings will be held with senior management of these organisations to explain the purpose of the study and the practicalities involved if the study were to be implemented within the organisation.

#### Recruitment of participants

Recruitment methods will include email invitation to employees and posters placed around each workplace advertising the study. Emails and posters will include the website address and a web-link will be added to the organisations’ intranet sites (previously tested in our pilot study [27]). Potential participants will be able to access further information (including the Participant Information Sheet) and register their interest to participate on the study website.

Potential participants will be asked to complete a screening questionnaire via the study website or by telephone, to confirm their eligibility, based on the following inclusion criteria: based at recruited worksite at least 4 h/day (within core hours of 8 am-6 pm) on at least 3 days/week; current contract lasts for duration of the study (i.e.18 months) (to exclude temporary workers); access to internet at work; able to give informed consent; able to communicate in English; no self-reported recent history of myocardial infarction or stroke or physical limitations that would limit ability to participate in physical activity (assessed using the Physical Activity Readiness Questionnaire) [32].

All participants who meet the eligibility criteria and consent to participate will be contacted by a member of the research team by telephone or email to complete the baseline assessment. Following this, clusters of participants will be randomised to the Intervention or Control Group using computer generated random numbers. Clusters have been defined as the smallest organisational unit (e.g. a department or office/floor) within each participating workplace.

### Randomisation

A random allocation sequence will be drawn up by the trial statistician and group allocation placed in sequentially numbered, sealed opaque envelopes. Group allocation will be stratified to ensure similar numbers of clusters in the Intervention and Control Groups in each participating organisation.

### Intervention

#### Intervention group

**Fig. 1:**
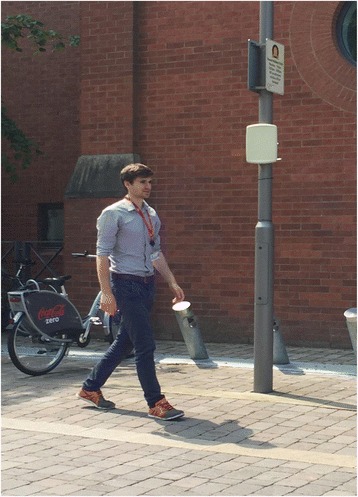
Physical activity monitoring system

The Physical Activity Loyalty (PAL) scheme is a complex multi-component intervention based on concepts similar to those that underpin a high-street loyalty card aimed at encouraging repeated behaviour (i.e. loyalty) [24] and is designed to incorporate a range of behaviour change techniques. Components include the provision of ‘points’ and rewards (financial incentives) contingent on meeting targeted physical activity behaviour goals (extrinsic motivation, goal-setting). Participants will be encouraged to undertake 150 mins/week of physical activity which is in line with current guidelines [33]. The PAL scheme integrates a novel physical activity tracking system with web-based monitoring and evidence-based behaviour change tools (i.e. self-monitoring, goal-setting). The proposed 6-month intervention will involve placing sensors (wifi beacons) in the vicinity of participating workplaces at specific locations to encourage physical activity within a 2 km radius of participants’ worksites (see Fig. 1) (i.e. provision of prompts/cues, habit formation, addition of objects to the environment). Locations include along footpaths, local parks, leisure centre, shopping mall, bus stops and train stations. Maps of various walking routes and details about physical activity opportunities tailored to the workplace will be provided on the study website (instruction on how to perform behaviour) [34]. Participant’s activity will be logged when they pass within an approximate 25 m radius of the wifi sensors with their PAL keyfob when undertaking physical activity (e.g. walking). This logs the place, date and time of the bout of activity. Participants can log onto their account on the study website and receive real-time feedback on minutes of their physical activity. Minutes will be converted to ‘points’ (10 ‘points’ for 1 min of activity recorded), and collected ‘points’ are redeemable for rewards (downloadable retail vouchers) sponsored by, and redeemable at, local businesses. To reduce the risk of ‘gaming’, a daily ‘points’ cap will be implemented and the transit times between sensors checked for anomalous values [27]. Bonus rewards and ‘Double Points Days’ will be offered when participants meet specific weekly physical activity targets. [17].

To tailor the intervention, a purposive sample of employees (both men and women across a range of ages) will participate in three focus groups (maximum 10/group) prior to the intervention to explore aspects such as the availability of, and preferences for opportunities for physical activity proximal to the workplace. Sensor locations will be determined from the feedback received in the focus groups regarding popular walking routes. Employees’ opinions on the website interface and the rewards redemption function, for example, will also be considered. To determine incentive levels we will use stated preferences of the participants from the DCEs to assess their mean Willingness to Accept (WTA), Willingness to Pay (WTP) and the trade-offs they would make for the attributes [35] of the incentive programme, prior to the intervention. This information will help us determine the level of the rewards available for earned ‘points’ (further detail below).

In addition to the financial incentive element, the intervention has several other components designed to enhance the effectiveness of the incentives as advocated by Marteau et al. [17]. These components are delivered via the study website and designed to have multiple effects: (a) to increase usage of the study website, (b) as effective Behaviour Change Techniques (BCTs) in their own right, and (c) as techniques designed to aid the transition from more extrinsically motivated behaviour to more intrinsically motivated habitual behaviour. The techniques include the provision of regular tailored motivational emails, tailored feedback, information on walking routes in the vicinity of the participating workplaces and links to other resources such as physical activity advice and healthy eating guidelines. It also includes self-regulation techniques of goal setting, self-monitoring, and prompts to behaviour [36].

#### Underpinning theoretical framework

**Fig. 2:**
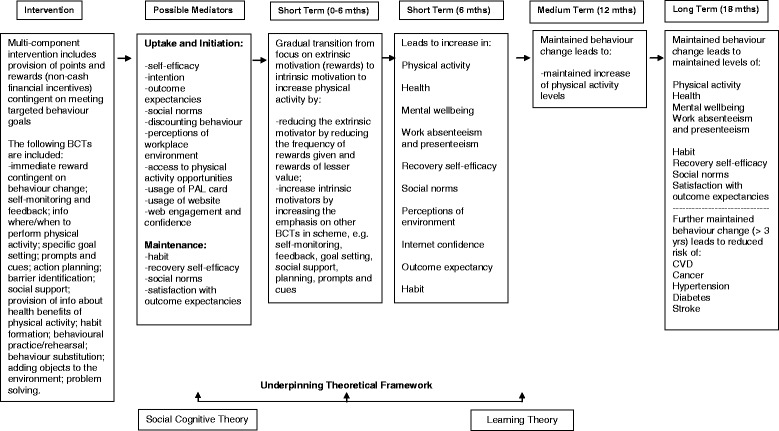
Logic Model of the Physical Activity Loyalty Scheme

The financial reward component of the intervention is based on principles of Learning Theory by providing an immediate reward (extrinsic motivation) for behaviours that offer health gains in the future. It also contains elements based on other approaches, such as goal setting, prompts, self-monitoring, and habit formation which fit within a self-regulation control theory framework [37], motivational messages (persuasion), and social support (vicarious experience) which should increase self-efficacy according to Social Cognitive Theory [38]. Social Cognitive Theory also holds that satisfaction with the consequences of behaviour change can act as a reinforcing mechanism, in addition to the reinforcement of financial rewards. Thus, the financial incentive component is embedded in a complex intervention containing evidence-informed BCTs, as previously advocated [17]. Figure 2 presents the logic model underpinning the intervention development.

#### Control group

Those assigned to the waiting-list control condition (n = 388) will be offered the opportunity to participate in the intervention after the 18-month follow-up period. Participants in this group will complete outcome measures at the same time ‘points’ as the Intervention Group.

### Outcome measures

All outcome measures will be analysed and, where required, collected by a Postdoctoral Research Fellow (PDRF) blinded to group allocation. Self-reported outcome measures will be collected at baseline, 6 months and 18 months (unless otherwise stated), via online questionnaires distributed by email and automatically collated via Qualtrics (www.Qualtrics.com) (successfully employed in our pilot trial [27]).

#### Physical activity

The primary outcome is mean steps/day objectively measured by a sealed pedometer (to blind participants to the output) worn on the waistband (Yamax Digiwalker CW-701, Japan), for which reliability and validity has been established [39–41]. Participants will wear the pedometer for 7 consecutive days, complete a wear time diary, and the Global Physical Activity Questionnaire (GPAQ) to elucidate the context of activity undertaken [42]. These measures will be collected at baseline, 6 (immediately post-intervention), 12 (6 months post-intervention) and 18 months (1 year post-intervention). This schedule ensures that we can account for the impact of seasonality of physical activity behaviours on the intervention effect.

#### Health and wellbeing

Secondary outcome measures of health (Short Form-8 (SF-8)) [43], Quality of Life (EQ-5D-5 L) [44–46], and well-being (Warwick-Edinburgh Mental Wellbeing Scale (WEMWBS)) [47, 48], are included. Items from the SF-8 questionnaire can be derived to give an indication of both physical and mental health. The WEMWBS comprises 14 positively worded statements, where scores are summed with higher scores indicating greater mental well-being. The EQ-5D-5 L questionnaire is a health state utility measure based on five dimensions of mobility, self-care, usual activities, pain/discomfort, and anxiety/depression, and a visual analogue scale (0–100) EQ-5D-5 L index-based values (utilities) derived from a crosswalk study are then applied to the EQ-5D-5 L levels to translate changes in health states to Quality Adjusted Life Years (QALYs) for use in cost-utility analysis [45].

#### Work-related impacts

Specific questions from the WHO Health and Work Performance Questionnaire [49] will be used to measure work presenteeism (measurable extent to which physical or psycho-social symptoms and conditions adversely affect the work productivity of those in work [50]). This validated method comprises three questions with answers on an 11-point Likert scale asking participants to rate their job performance levels, and has been demonstrated to be sensitive for detecting change following a physical activity intervention [51]. Work absenteeism will be measured by asking participants to state the number of day’s sick leave in the past 6 months and will be verified objectively by each organisations’ Human Resource Department where possible.

#### Mediators/moderators

We will collect data that are hypothesised to mediate loyalty scheme use, initiation (4 weeks) and maintenance of physical activity behaviour (6 months post-baseline). It is hypothesised that change in mediators should be apparent at 4–6 weeks and that mediators of early behaviour change are distinct from those affecting maintenance [52–54]. These theoretical constructs and associated outcome measures have been informed from the mapping of BCT’s from the pilot study and evidence from the relevant literature on behavioural initiation and maintenance [55–60]. Common core theoretical constructs of behaviour change including outcome expectancy [61], self-efficacy [62] and intention [63], motivation, financial motivation, planning, subjective norms, stated WTP and WTA derived from the DCEs, will be collected at baseline and 4 weeks to assess incentives to initiate behaviour change. Subjective social network data will be collected by work colleague nominations, with participants asked to nominate up to five colleagues that they usually socialise with and would also partake in physical activity with at baseline and, 6 and 18-months follow-up. Furthermore, social networks will be inferred when user’s keyfobs are “sensed” at sensors placed at physical activity opportunities in the vicinity of participating workplaces [64]. Perceptions of workplace environment [65] and objective measures of the workplace environment using Geographical Information Systems (GIS) data including walkability [66], street connectivity, access to physical activity opportunities (shops, parks, leisure facilities, train/bus stations) will be measured [67] at baseline. Other measures of web engagement, confidence in using the internet and loyalty scheme usage which may mediate intervention “dose” [68] and therefore behaviour change have been included.

To assess mediators of behaviour change maintenance, habit using the Self-Report Habit Index [69], recovery and maintenance self-efficacy [70], social norms [71], satisfaction with outcome expectancies [61], motivation, planning and social support, stated WTP and WTA, will be captured at baseline and 6 months. Data on inter-temporal and risk preferences will be collected at 6 months only (immediate post-intervention). In addition, subjective social network data will be collected at 6 and 18 months.

#### Compensatory behaviours

To assess the impact of the intervention on compensatory behaviours, data on smoking, alcohol and short Food Frequency Questionnaire (FFQ) [72] will be collected.

#### Economic evaluation

The primary economic evaluation will take the form of a within trial cost-utility and cost-effectiveness analysis, with health outcomes expressed in terms of Quality-adjusted Life Years (QALYs). Changes in health-related Quality of Life (as expressed using QALYs using EQ-5D data) will be measured from the participant’s perspective. Utilisation of healthcare resources will be captured using a specially devised online health and social care resource use data collection form. Intervention costs will be obtained using a modified template [73], explicitly discriminating between intervention and research costs.

Revealed preferences [74] and stated preferences (i.e. DCE [75]) questions will be used to gain information on the optimal level of financial incentives necessary to trigger behaviour change. Revealed preferences questions will investigate the current amount of time participants spend on activities including sleeping, leisure, work, transportation, home, physical activity and sedentary activity. The key behavioural decisions in this model are the distinct but related decisions to participate in physical activity and duration of each session of activity [76]. We will then investigate how participants’ wages, hours worked, fixed costs of physical activity (e.g. gym membership fees), and variable costs of physical activity, affect the amount of time participants spend doing physical activity [77, 78] to assess how income and substitution effects affect physical activity. Stated preferences questions and DCE questions will investigate participants’ WTA for physical activity, and the rate of trade-offs between physical activity, other time-related activities and money [79] to estimate the optimal mean level of the financial incentives that would trigger behaviour change. Information on participants’ discount rates WTA for behaviour change, and behaviour change rates will provide an understanding on how the incentive alters the costs and benefits of this healthy behaviour and why it successfully results in changed behaviour in some participants and not others.

After the intervention (at 6 months), a random sample of 200 participants will participate in a behavioural economic field experiment aimed at eliciting individuals’ inter-temporal preferences and discount rates to identify participants who exhibit exponential discounting, hyperbolic discounting or quasi-hyperbolic discounting [80]. This will help us investigate whether there is a relationship between behaviour change, discounting, and financial incentives. Briefly, the behavioural economic field experiment will be conducted in maximum groups of 20 participants during lunchtimes. Participants will be offered a £20 gift voucher for taking part in the behavioural economic field experiment. Participants will face several dichotomous decision tasks, such as a risk preference choice task and a monetary discount rate choice task. After the choice tasks, the participant will randomly select the choice occasion and the alternative that will count for payment, and will throw a 10-sided die to determine whether the final payment will take place.

#### Process evaluation

Evaluation will employ a triangulated design using both quantitative and qualitative data. The process evaluation is concerned with five core research questions: i) What was participants’ exposure to the intervention? ii) To what extent was the intervention implemented across the participating organisations? iii) How, for whom and under what circumstances does the intervention bring about behaviour change? iv) How, for whom and under what circumstances does the intervention maintain behaviour change?; (v) Whether there were any unintended consequences of the intervention. The process evaluation will encompass the following:

#### Study context

A recording of current health improvement programmes and policies in each participating organisation will be collected throughout the duration of the study period. This will be supplemented by environmental measures, (such as distance to the nearest green space and neighbourhood walkability) and free car parking at work collected at baseline, which will assess the extent of physical activity opportunities for each participating organisation.

#### Intervention fidelity and “dose”

Fidelity of the intervention will be supported by the use of standardised training manuals and training sessions for those assisting with intervention delivery which includes the collection of the primary outcome data (pedometers) and all email communication with participants. They will be asked to keep a daily record of any problems with implementation using a customised proforma, and report to the project manager on a weekly basis throughout the 6-month intervention period. The research team will assess delivery fidelity using a quality assurance form. Weekly feedback on fidelity will be given to those involved in the delivery of the intervention to assist standardisation and completeness. Establishing intervention “dose” will draw on data regarding PAL scheme usage (minutes of physical activity/week recorded) and website exposure (e.g. frequency of visits; number of hits; number of visitors that accessed specific website content; mean duration of visits). These data will automatically be collected throughout the 6-month intervention. Extent of internet use will be assessed by asking participants how many hours per week on average they spend on the internet and to rate their confidence on using the internet on a 10-point likert scale [68]. In addition, information on those who redeemed their ‘points’ for rewards and who subsequently reimbursed their rewards at nominated retailers will be collected. Compliance with the intervention will be monitored via the PAL scheme usage data, objectively recorded using the tracking system, and the use of the web resources as outlined above.

#### Participation and reach

Participation will be assessed by collating the actual number of participants recruited versus the number invited to participate. Reach will be assessed by investigating the representativeness of study participants in regards to gender, age, ethnicity, socio-economic position (SEP) (compared to aggregate demographics of workforce in participating departments).

#### Responsiveness

We will assess the experiences of participation, aspects of acceptability for those who engaged versus those who didn’t, and any barriers or facilitators to this. This will draw on exit questionnaires completed at 6 months to assess level of engagement and focus groups at 6 months (n = 5) which will explore their experiences of the intervention and determine the types of participants who benefitted from the intervention, how and why it worked for them. We will purposively sample participants (maximum n = 10/group) to ensure a representative sample, including those actively engaged in the intervention and those not (i.e. dropped out) are recruited. Focus groups will be repeated at 18 months to ascertain the views of those who have maintained behaviour change and those who have not, and why. A schedule of open-ended questions will be used to elicit information about reactions to the intervention; barriers to physical activity and; suggestions for future roll-out of the intervention if proven effective. The final focus group will seek confirmation of the results from the previous focus groups via triangulation of the data. Semi-structured interviews with senior managers of participating employers (n = 7) and participating retailers (n = 5) will be used to explore their perceptions of being involved in the study.

#### RE-AIM framework

All data will be interpreted in the context of the RE-AIM framework [81].

This will ensure that we have a clearer understanding of the **R**each, **E**ffectiveness, **A**doption, **I**mplementation and **M**aintenance. This framework allows concurrent evaluation of dimensions considered relevant to ‘real world’ implementation, such as the capacity to reach target population and to change physical activity. In particular, we will examine differences across social groups and whether the intervention has impacted on inequalities in physical activity participation in the study population.

### Statistical analysis

For the primary analysis, mean steps per day at 6 months will be the dependent variable. A random intercept will be fitted at the cluster level (all other variables will be fixed effects), with group, organisation (in categories), and baseline mean steps per day added to the model (Objective 1). The main focus for the analysis will be the estimated coefficient representing the difference in mean steps per day between the Intervention and Control Group, adjusted for baseline differences. The model will then be extended through the inclusion of interactions involving group and relevant covariates to test for any differential effects of the intervention (e.g. age, sex, SEP, time discounting function). In light of the multiple testing involved, these subgroup analyses will be cautiously reported as hypothesis generating rather than confirmatory.

The primary analysis will then be repeated using 18-month follow-up data to investigate the effectiveness of the intervention for behaviour change maintenance (Objective 2). These analyses will be repeated with secondary health and wellbeing outcomes, and work-related outcomes (Objective 4 and 5). Further, sensitivity analyses will assess the impact of missing data using Multiple Imputation by Chained Equations (MICE), testing first whether missingness-at-random is plausible [82].

#### Mediation analyses

Mediation analyses of early behaviour change (uptake and initiation) (4 weeks) and behaviour change maintenance (6 months from start of intervention and 12 months from cessation of intervention) will be conducted (Objective 6). Methods for assessing and estimating the direct/indirect effects of intervention in the presence of multiple mediator models will be employed [83]. Bootstrap re-sampling strategies will be used to avoid multivariate normality assumptions in placing confidence limits on the estimates of the proportion of the total intervention effect that is mediated [84]. However, these analyses must be interpreted cautiously given the limitation that the mediation model is assumed to be correctly specified both in functional form and in that no variables are omitted which affect the relationships between independent and mediator or between mediator and dependent variables.

Briefly, social networks will be constructed from the work colleague nomination data at baseline, 6 and 18 months using UCINet 6.9 [85]. These data will be supplemented by objective, real-time proximity data collected throughout the 6 month intervention period. Individual-level network variables, for example, density and centrality measures, will be derived and included in mediation analyses. Further analyses investigating the structure, characteristics, function and evolution of workplace social networks on physical activity behaviour change will be undertaken as part of a NIHR Career Development Fellowship.

#### Economic analyses

The economic evaluation will comprise a cost-effectiveness and cost-utility analysis from the public sector perspective and a cost-benefit type analysis from the employer’s perspective (Objective 3). A cost-effectiveness and cost-utility analysis will compare costs and outcomes associated with the Intervention to those associated with the Control Group at three time-points. For the initial within study analysis, the outcome measures used will be (a) changes in mean steps per day; and (b) a within study measure of QALYs determined from EQ-5D. QALYs will be adjusted for any imbalances between groups at baseline [86]. Thus, the initial analysis will present an estimate of cost-effectiveness of the intervention in terms of the incremental costs associated with increasing physical activity and a cost-utility analysis in terms of incremental costs per QALY gained. The uncertainty surrounding the estimates of cost and effects for the Intervention and Control Group will be investigated through the use of bootstrapping [87]. In a sensitivity analysis we will compare subgroups and assess the uncertainty in their incremental cost-effectiveness ratios (ICERs) by plotting the associated cost-effectiveness acceptability curves. The longer term analysis will employ a decision model, populated with reference to the literature, to link short term study outcomes to longer term impacts on health and wellbeing. The model structure will be informed by a review of other models undertaken in this area, including the modelling work undertaken for NICE (2008) [88]. Data will be embodied in the model through the specification of probability distributions for each parameter, to reflect the uncertainty. Probabilistic sensitivity analysis will be undertaken, using Monte Carlo simulation, to investigate the uncertainty surrounding the longer term estimates of costs, effects and cost-effectiveness of the intervention.

From the public sector perspective, the analysis will involve firstly generating ICERs using healthcare utilisation data and EQ-5D data. Secondly, from the employer’s perspective, the effect of physical activity on absenteeism will be used to estimate potential cost savings to employers (absenteeism model); and thirdly, we will employ an algorithm to translate changes in EQ-5D into quantifiable changes in productivity (productivity model [89–91]). Individual EQ-5D scores at baseline, 6 and 18 months will also be converted into estimated levels of productivity using this algorithm and the total productivity gain over 6 and 18 months for each group will be calculated. To calculate the ICER, from an employer’s perspective, the additional costs will be divided by the additional gain in total employee productivity by the Intervention Group.

#### Analysis of discrete choice and economic experiments (objective 8 and 9)

To analyse the revealed preferences data we will use a two-step modelling approach to explain an individual’s physical activity in relation to his/her characteristics [73, 74]. The revealed preferences data will be analysed with a Cragg’s double hurdle model [92–94] to first address physical activity participation, and then to analyse the amount of physical activity. We will run separate models for different types of physical activity (e.g. walking, gym) to produce estimates for the effect of wage, cost of physical activity and participants’ characteristics on the amount of physical activity. This model will provide us with a ‘revealed’ implicit monetary value for units of physical activity. A random utility econometric model will be fitted to the DCE data to determine the implied individual thresholds inducing behaviour change [80]. Estimation will take place in a panel specification, and Bayesian posterior estimates of threshold values will be informed by the sequence of responses. Data from the field experiment to assess time and risk preferences will be analysed using maximum likelihood estimates, allowing for within-subject clustered standard errors, as each participant answers more than one time preference choice [76]. The data collected on the amount of physical activity at baseline and after the intervention will allow us to run another Cragg’s double hurdle model for behaviour change, and explore how differences in inter-temporal preferences, baseline physical activity levels, incentives and other participant’s characteristics affect behaviour change. The Cragg’s double hurdle model will allow us to investigate the two-step process in behaviour change: firstly, relating to the decision to do more physical activity compared to baseline; secondly, the decision of how much physical activity to do.

#### Process evaluation analyses

Focus groups and semi-structured interviews will be audio-recorded and analysed using thematic content analysis [95] whereby identified themes will be represented in a matrix for further analysis and interpretation [96, 97] (Objective 7). Qualitative data will be entered into NVivo, which will be used to manage the relevant data and to explore inter-relationships between themes. Each transcript will be coded independently by two researchers. Analysis will explore implementation and receipt of rewards and contextual factors affecting these. Potential causal pathways will be explored in order to develop hypotheses relevant to secondary moderator and mediator analyses. Additionally, quantitative data from PAL scheme usage and web usage will be used in the analyses of intervention fidelity, using simple descriptive statistics. Within the context of the RE-AIM Framework, we will compare the characteristics of our trial population with the target population, to gauge the potential generalisability and impact of our results. Propensity scores will be used to compare the characteristics of trial participants with the inactive working population as a whole in Northern Ireland, using available population-level data [98]. These propensity scores will also be used as to ascertain whether a weighting method can be employed, using the scores, to generalise the results to the target population.

### Update on study progress

During the recruitment phase for the study, a revised power calculation was undertaken assuming a less demanding effect size estimate taken from the recent literature together with our actual baseline data on mean and variance of cluster size and an intra-class correlation co-efficient of 0.029. In the original protocol, the sample size calculation for the trial was determined using an anticipated effect size of d = 0.21 (based upon a previous meta-analysis of workplace based physical activity interventions). However none of the included studies were incentive-based interventions for physical activity. More recent literature has been published [99, 100], including a meta-analysis in which a mean effect size for incentive based interventions of 16 min of MVPA per day was estimated, equating to an effect size of approximately 1600 steps (d = 0.40). Additionally, in the TRIPPA study [100], which is examining the influence of financial incentives on the effectiveness of a wireless-upload pedometer to encourage weekly physical activity goals, the study was powered to detect a difference of a minimum of 30 min of MVPA per week between groups and power calculations were based on considerably higher effect size than assumed in our original protocol (0.35). Therefore, under the assumption that our original estimate was too conservative, the power calculation has been updated. For an effect size of 0.40, a study of 330 per group (or 660 in total) would have 90 % power at the 5 % significance level. Assuming a 15 % drop-out, the study would need to randomise 776 participants.

## Discussion

Given the rise in physical inactivity and associated health conditions, such as diabetes, heart diseases and cancer, worldwide, there is a growing need for scalable, effective, and affordable public health interventions. This study will provide necessary research concerning the potential effectiveness and cost-effectiveness of a novel workplace physical activity intervention and an understanding of assumed pathways of behaviour change initiation and maintenance. Further, detailed cost-effectiveness analyses will assess whether financial incentives are an appropriate use of government and private sector resources. The scope for introducing financial incentives in public health is extensive, but there is much to learn.

The study is designed to maximise the potential for roll-out in similar settings, by engaging the public sector and business community in designing and delivering the intervention. In collaboration with retail partners, an existing “proven” mechanism that delivers behaviour change in the private sector was applied to a public health setting using a sustainable “Physical Activity Loyalty Card.” We have developed a sustainable business model using a ‘points’ based loyalty platform, whereby local businesses ‘sponsor’ the incentive (retail vouchers) in return for increased footfall to their business, which is aligned to precepts of the Department of Health Public Health Responsibility Deal [13]. For financial incentive schemes to be worthwhile in the longer term, they must be based on a sustainable model. The UK Government and the U.S. government have encouraged those involved in public health to work collaboratively with business. This study provides an example of how researchers can successfully engage with the business sector in public health.

## Abbreviations

BCTs, behaviour change techniques; BMI, body mass index; DCE, discrete choice experiment; EQ-5D-5 L, euro-Qol 5 dimension 5 level questionnaire; FFQ, food frequency questionnaire; GIS, geographical information systems; GPAQ, global physical activity questionnaire; ICER, incremental cost-effective ratio; Km, kilometres; m, metres; MICE, missing imputation by chain equations; MRC, medical research council; MVPA, moderate-vigorous physical activity; NHS, national health service; NICE, national institute for health and care excellence; NIHR, national institute for health research; PAL, physical activity loyalty; QALY, quality adjusted life year; RCT, randomised controlled trial; RE-AIM, reach, effectiveness, adoption, implementation, maintenance; SD, standard deviation; SEP, socio-economic position; SF-8, short form-8; SPIRIT, standard protocol items: recommendations for intervention trials; TRIPPA, trial of economic incentives to promote physical activity; UK, United Kingdom; US, United States; WEMWBS, Warwick Edinburgh mental wellbeing scale; WHO, World health organisation; WTA, willingness to accept; WTP, willingness to pay

## Additional file

Additional file 1: Web platform for physical activity web-based interventions. (PDF 1121 kb)
