# Supplementary material for: Effectiveness and cost-effectiveness of a physical activity loyalty scheme for behaviour change maintenance: a cluster randomised controlled trial
Source: BMC Public Health. 2016 Jul 22;16:618. doi: 10.1186/s12889-016-3244-1 (PMC4957294; doi:10.1186/s12889-016-3244-1)
Supplement: Additional file 1: — Web platform for physical activity web-based interventions. (PDF 1121 kb) [file 12889_2016_3244_MOESM1_ESM.pdf]

Queen's University Belfast

The Centre of Excellence for Public Health Northern Ireland

Open source platform to develop, implement and evaluate web-based physical activity interventions

We have developed an open source platform that other researchers can adapt for physical activity web-based interventions

The web-based platform consists of 3 main components:

- An outward facing website which contains health promotion material, behaviour change tools, integrated social media, and mechanisms for automated motivational messages;
- A research tool which consists of modules for screening, recruitment, electronic data collection and reporting; and,
- Integration of the system with other technologies for monitoring physical activity, for example, FitBit devices.

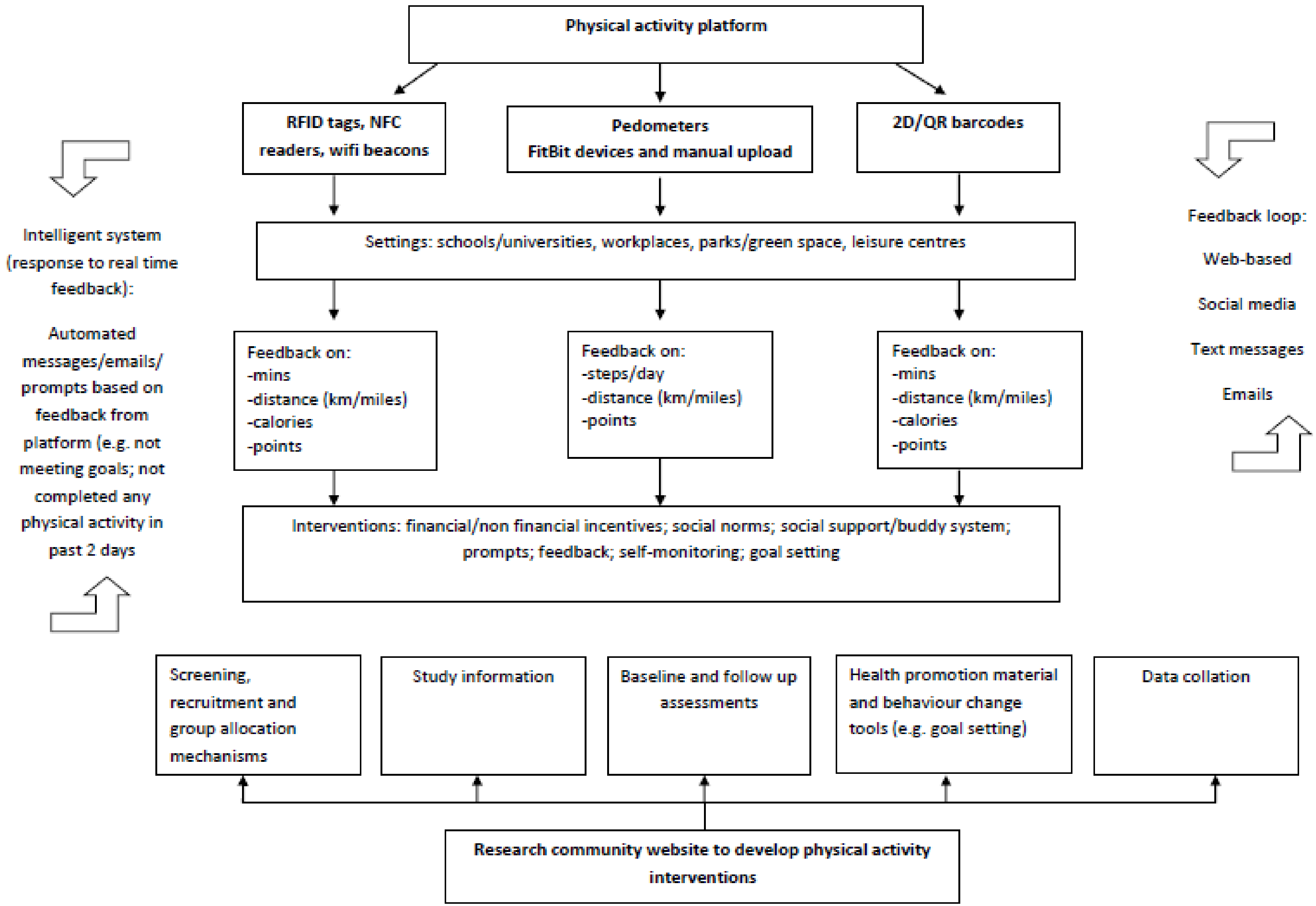
Physical activity platform
RFID tags, NFC readers, wifi beacons
Pedometers FitBit devices and manual upload
2D/QR barcodes
Settings: schools/universities, workplaces, parks/green space, leisure centres
Feedback on: -mins -distance (km/miles) -calories -points
Feedback on: -steps/day -distance (km/miles) -points
Feedback on: -mins -distance (km/miles) -calories -points
Interventions: financial/non financial incentives; social norms; social support/buddy system; prompts; feedback; self-monitoring; goal setting
Intelligent system (response to real time feedback):
Automated messages/emails/ prompts based on feedback from platform (e.g. not meeting goals; not completed any physical activity in past 2 days
Feedback loop:
Web-based
Social media
Text messages
Emails
Screening, recruitment and group allocation mechanisms
Study information
Baseline and follow up assessments
Health promotion material and behaviour change tools (e.g. goal setting)
Data collation
Research community website to develop physical activity interventions

Research-specific Tools

- Recruitment and registration
- Screening
- Participant Info Sheet and consent
- Group allocation
- Data collection – online questionnaires
- Data collation and aggregation

Recruitment and Registration

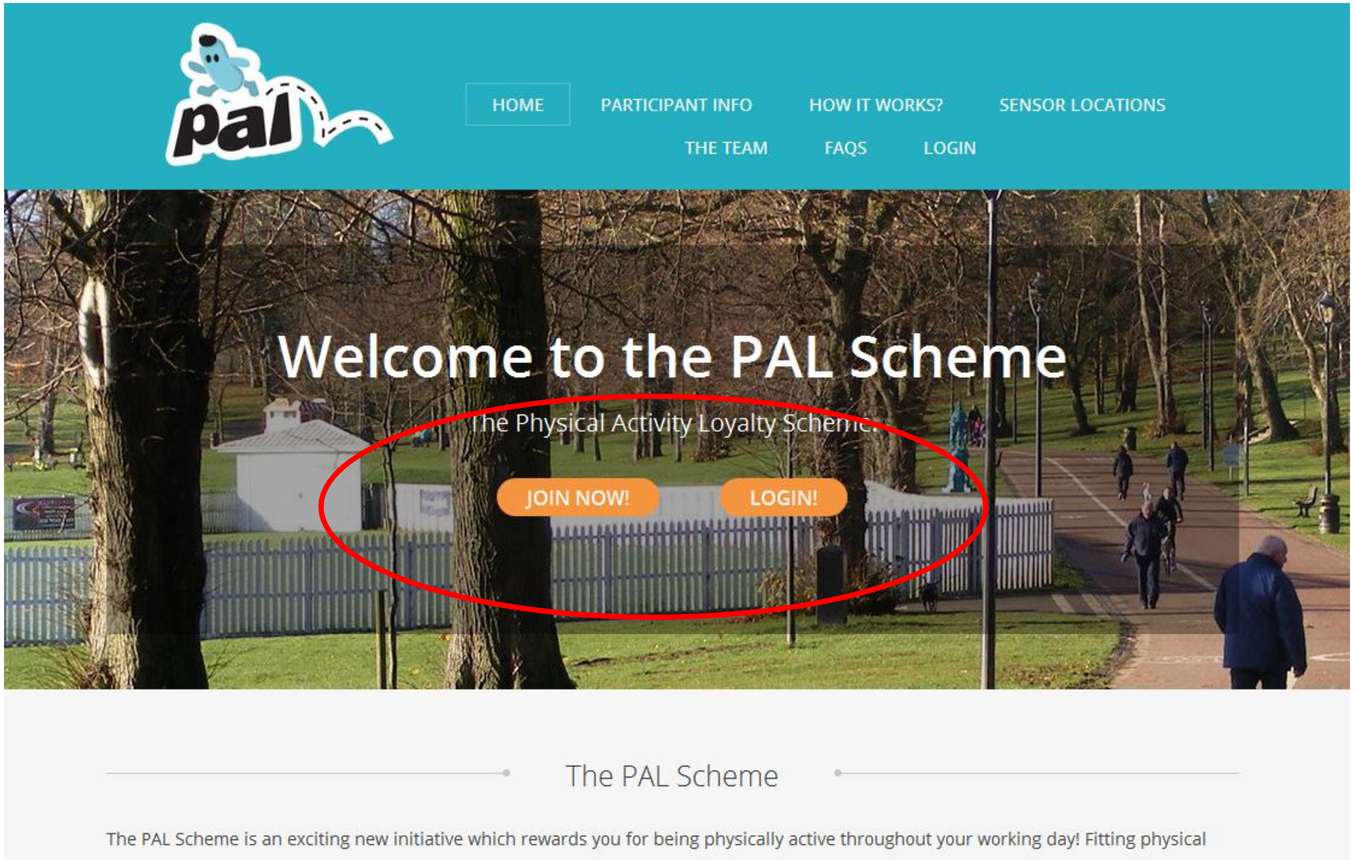

Participants are sent an email inviting them to participate in the study. This contains a web-link directing them to the study website where they can find out more information about the study and read the Participant Info Sheet. If interested, they can then register for the study by clicking "Register for the scheme" where they will be asked to complete their contact details and a short online screening questionnaire.

Screening

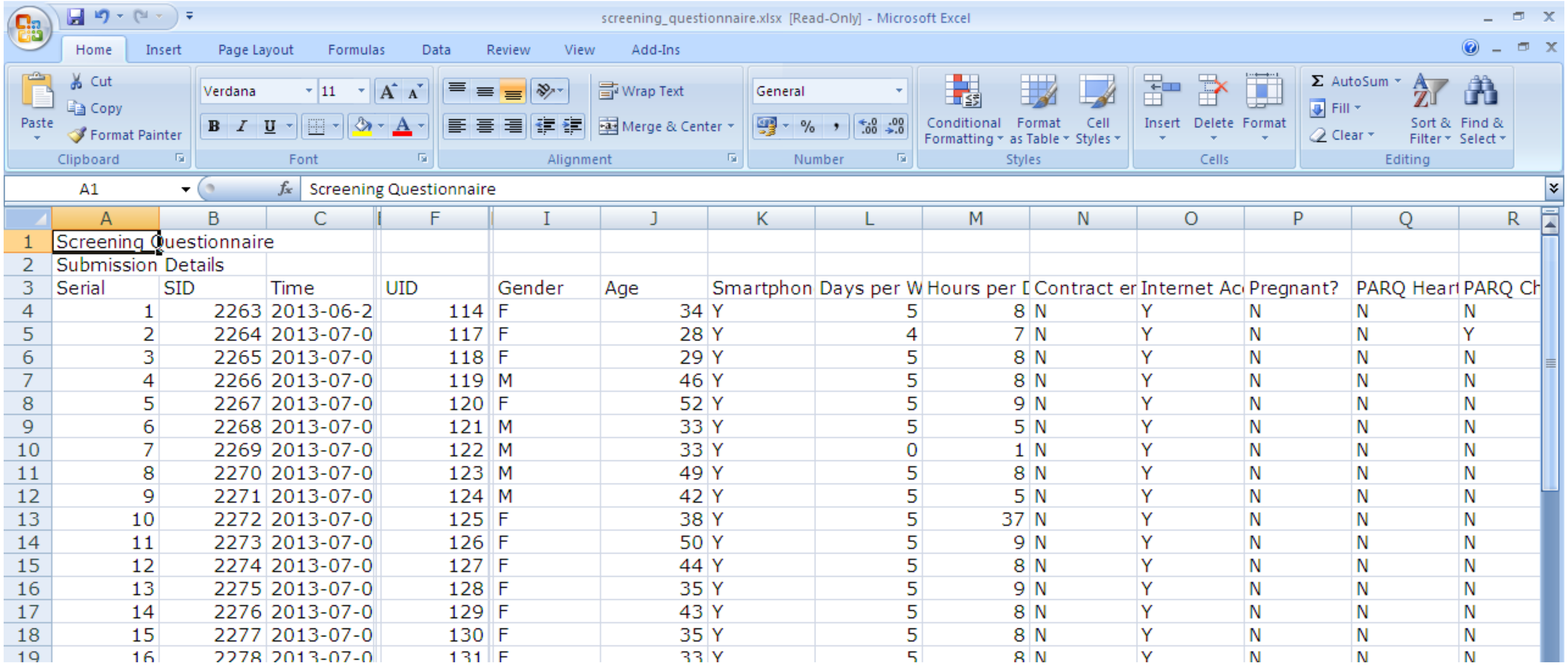

	A	B	C	F	I	J	K	L	M	N	O	P	Q	R
1	Screening Questionnaire													
2	Submission Details													
3	Serial	SID	Time	UID	Gender	Age	Smartphon	Days per W	Hours per [	Contract er	Internet Ac	Pregnant?	PARQ Hear	PARQ Ch
4	1	2263	2013-06-2	114	F	34	Y	5	8	N	Y	N	N	N
5	2	2264	2013-07-0	117	F	28	Y	4	7	N	Y	N	N	Y
6	3	2265	2013-07-0	118	F	29	Y	5	8	N	Y	N	N	N
7	4	2266	2013-07-0	119	M	46	Y	5	8	N	Y	N	N	N
8	5	2267	2013-07-0	120	F	52	Y	5	9	N	Y	N	N	N
9	6	2268	2013-07-0	121	M	33	Y	5	5	N	Y	N	N	N
10	7	2269	2013-07-0	122	M	33	Y	0	1	N	Y	N	N	N
11	8	2270	2013-07-0	123	M	49	Y	5	8	N	Y	N	N	N
12	9	2271	2013-07-0	124	M	42	Y	5	5	N	Y	N	N	N
13	10	2272	2013-07-0	125	F	38	Y	5	37	N	Y	N	N	N
14	11	2273	2013-07-0	126	F	50	Y	5	9	N	Y	N	N	N
15	12	2274	2013-07-0	127	F	44	Y	5	8	N	Y	N	N	N
16	13	2275	2013-07-0	128	F	35	Y	5	9	N	Y	N	N	N
17	14	2276	2013-07-0	129	F	43	Y	5	8	N	Y	N	N	N
18	15	2277	2013-07-0	130	F	35	Y	5	8	N	Y	N	N	N
19	16	2278	2013-07-0	131	F	33	Y	5	8	N	Y	N	N	N

Participants complete an online screening questionnaire. Researchers can automatically download the results of the screening questionnaire (in Excel format) to assess for eligibility. Rules can be set that automatically decide whether a participant is eligible for the study or not. Dependent on the outcome, participants can then be sent a tailored email advising them of the next step in the study or thanking them for their participation but unfortunately (for a certain reason) they are not eligible to take part.

Participant Information Sheet

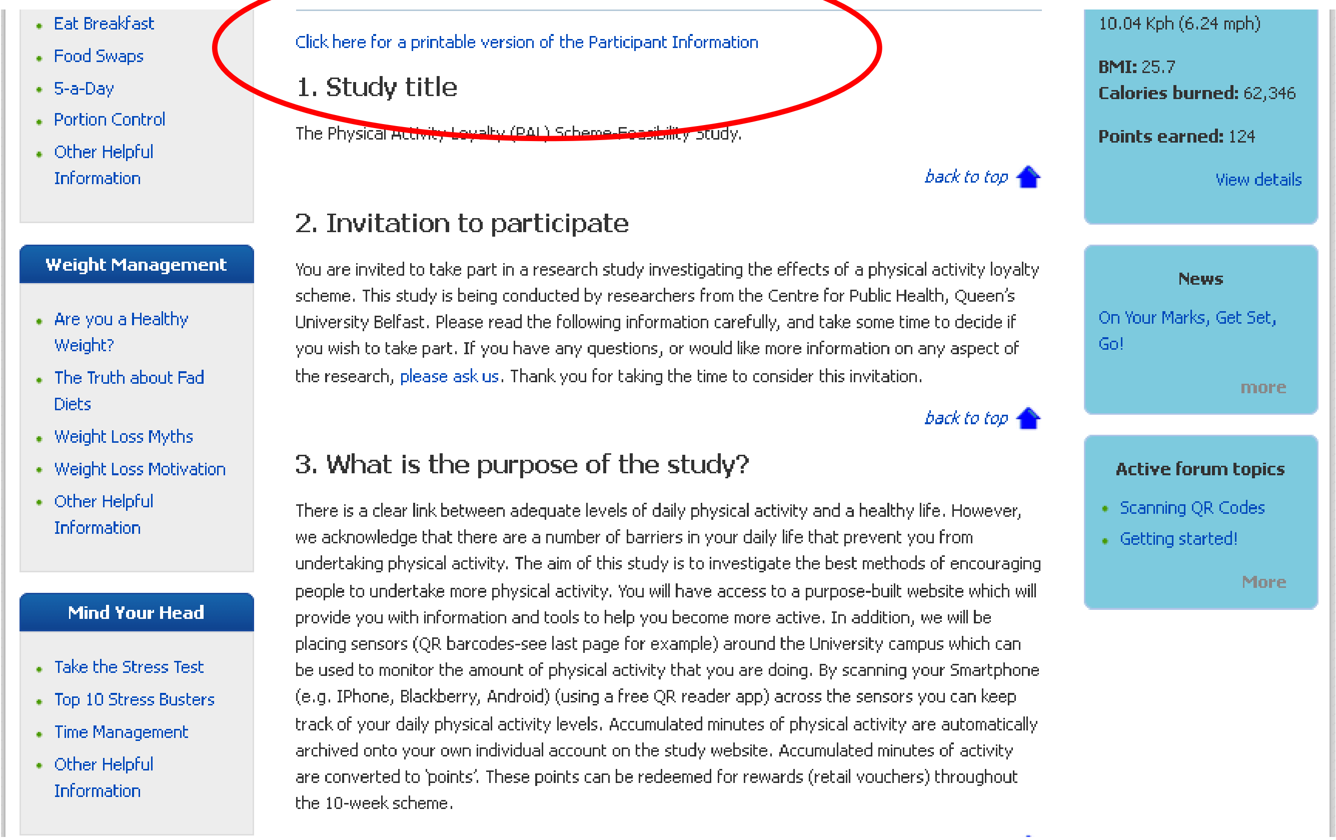

Participants can view the Participant Information Sheet on the website and also download a pdf copy. This can be easily accessed from a tab on the homepage. Other features include tabs on "how it works", "FAQ's" and a "contact us" feature to enable researchers to answer any questions participants may have.

Consent Form

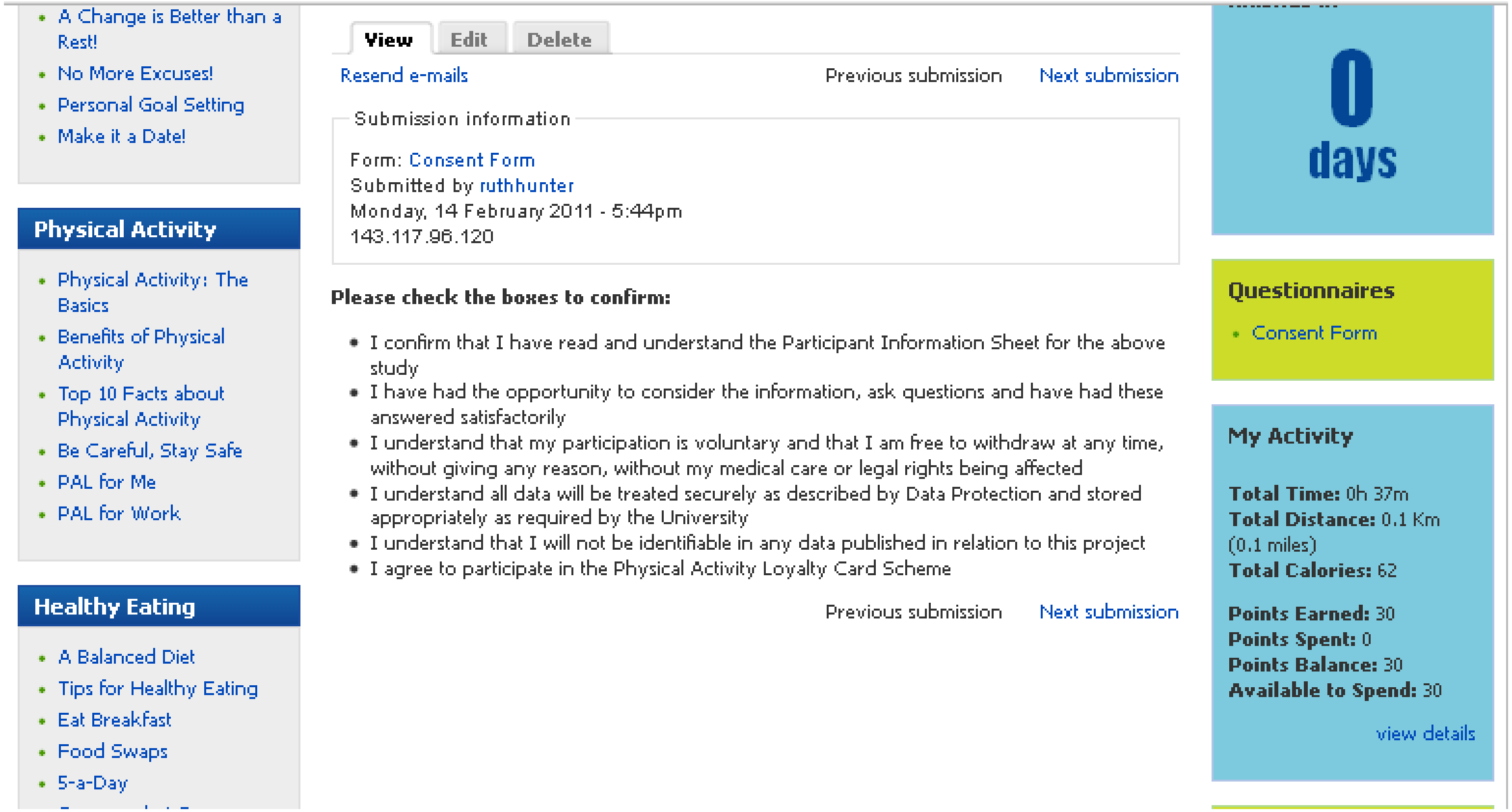

Participants can complete the consent form by checking boxes to confirm that they agree with each statement. This information is then collated on the back-end of the website along with the participant ID and date/time when the consent form was completed. Alternatively, participants can download a pdf copy of the consent form off the website, complete in written form and return via post/email.

Group Allocation

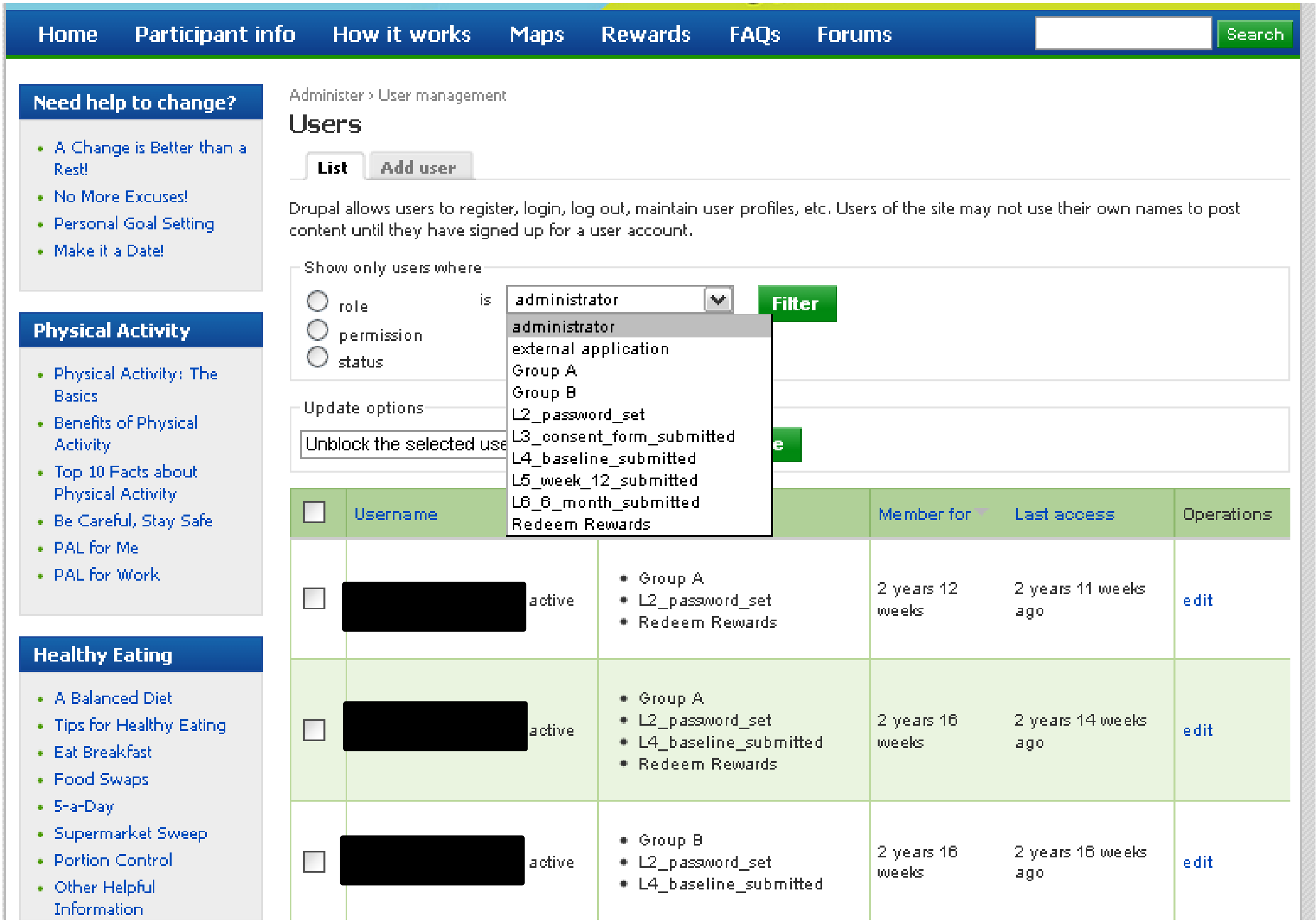

Researchers can assign participants to different groups on the website. This feature can allow Groups to be assigned different levels of access to features on the website. For example, those in the No Incentive Group were unable to access the Rewards page. The same feature can be used to assign different levels of access for pupils, parents and teachers.

Data Collection

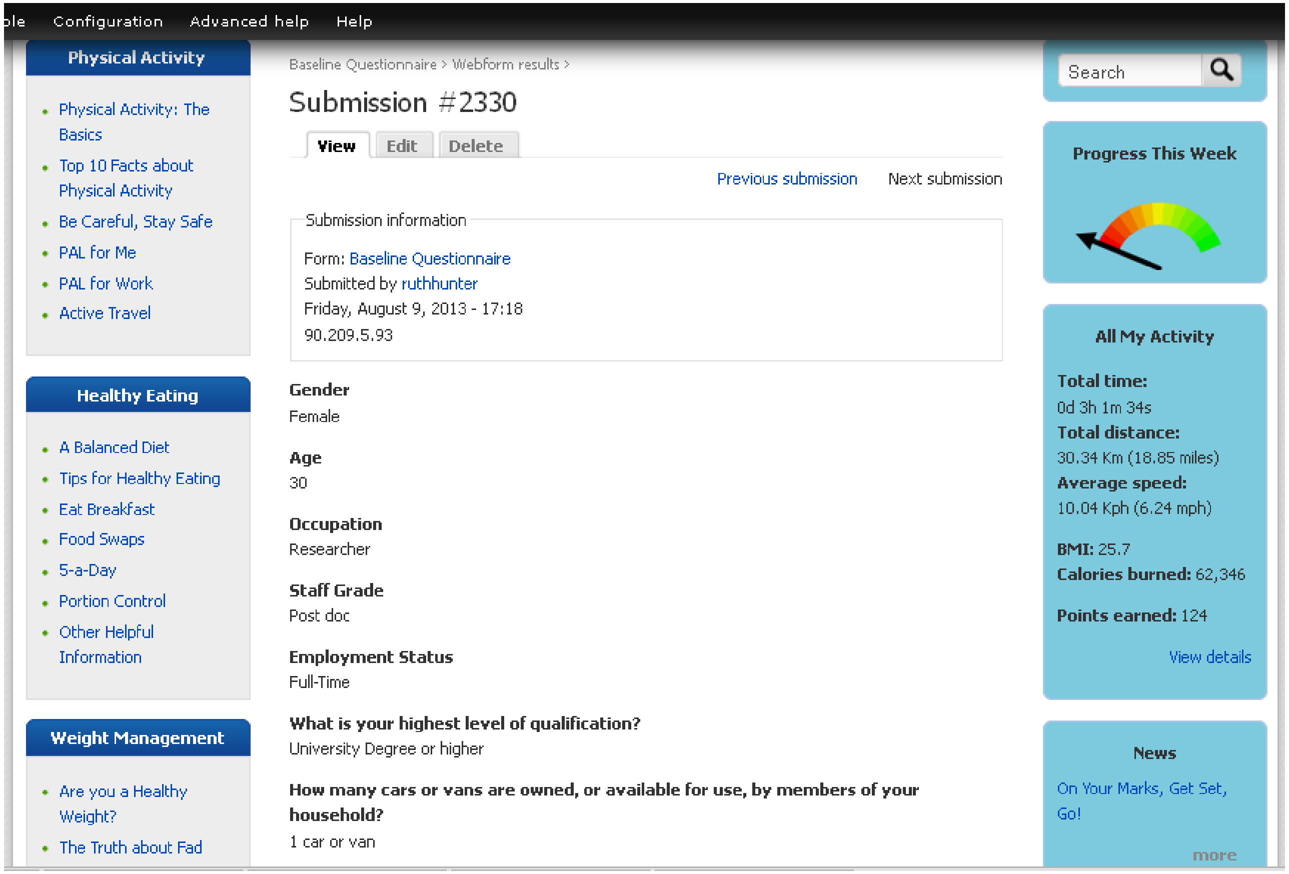

Participants can complete online questionnaires at each follow-up time point. The website can be programmed to send participants an automated email when these are due for completion. Similar to the screening questionnaire, results are collated on the back-end of the website and downloaded (on Excel spreadsheet) which can then be exported to SPSS etc for analysis.

Intervention-specific Tools

- Health promotion material
- Goal setting
- Planning
- Barrier identification
- Messaging and prompts
- Self-monitoring and feedback
- Social norms
- Incentives/rewards
- Tailoring physical activity opportunities to the local environment
- Social networks

Health Promotion Material

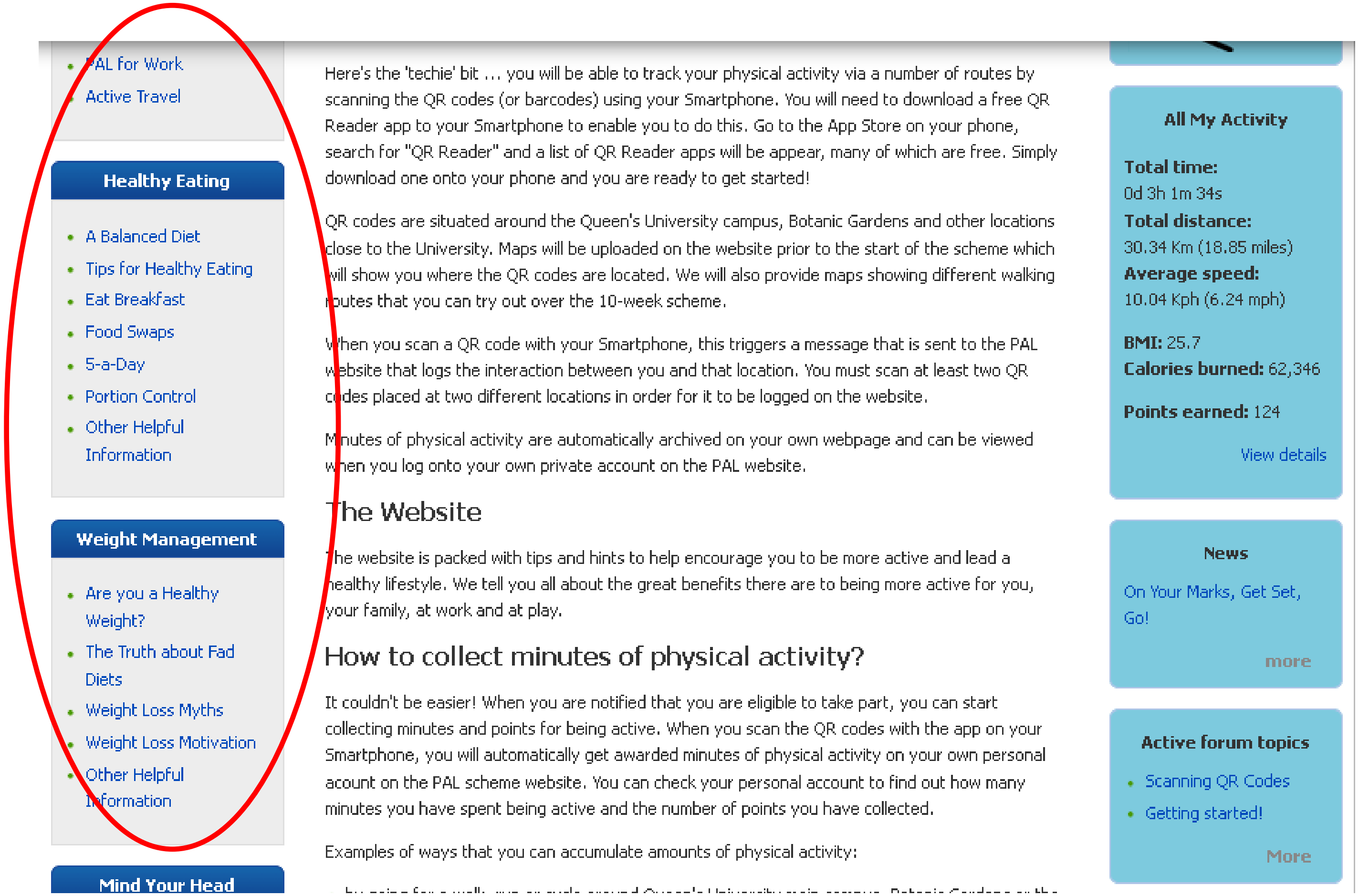

Participants can access general health promotion material for physical activity, diet, weight management, stress management , smoking , alcohol etc. This includes text, weblinks to further relevant resources and downloadable pdfs. Similarly, features exist for goal-setting, planning, barrier identification, automated messaging and prompts (via email, text, pop-up message) which can be linked to participant physical activity levels.

Self-monitoring and Feedback

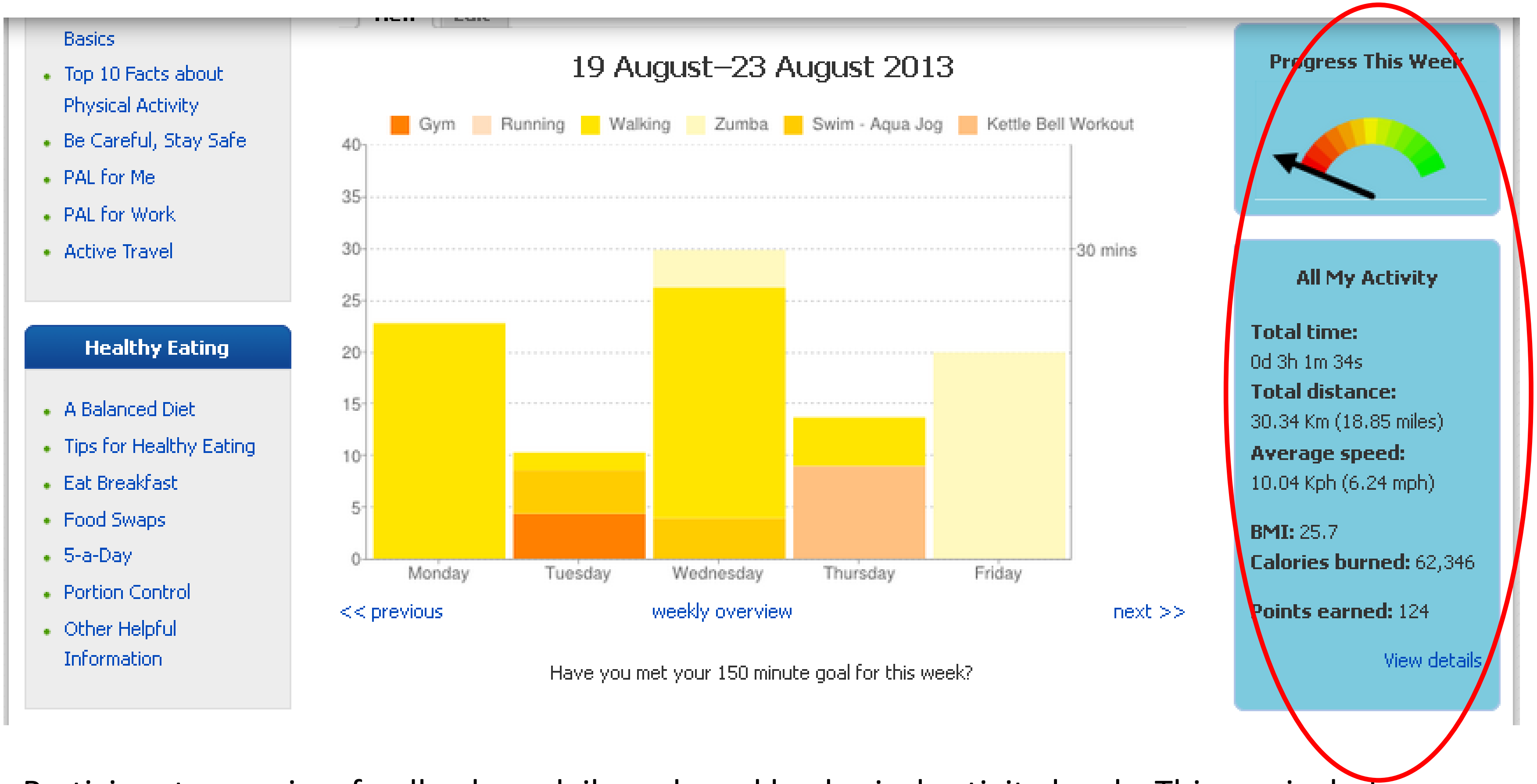

Participants can view feedback on daily and weekly physical activity levels. This can include minutes of activity, number of steps/day, distance , calories etc. Feedback can also be broken down to show different types of activity, for example, walking, running, gym, swim and exercise classes. The side panel shows an overall summary of total activity and a dial showing progress towards their weekly goal.

The platform also contains functionality to enable different types of feedback, for example, on social norms and competition.

Incentives/rewards

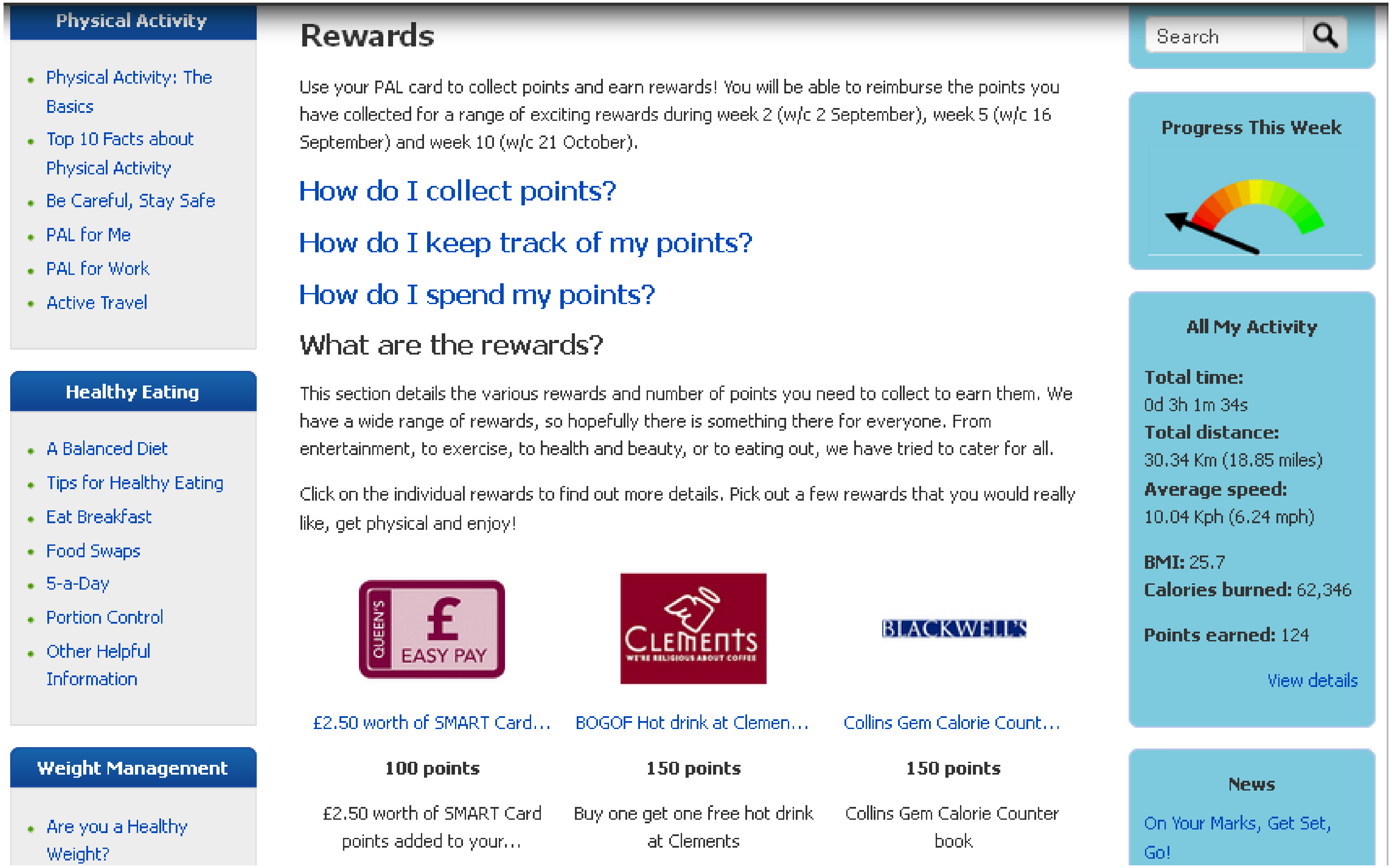

Participants were awarded points for minutes of activity. These could then be reimbursed for various rewards (retail vouchers). The website showed the rewards that were available and contains a mechanism where participants can spend/chose their rewards online. This information is then collated on the back-end where researchers can view what rewards participants have chosen.

Tailoring physical activity opportunities to the local environment

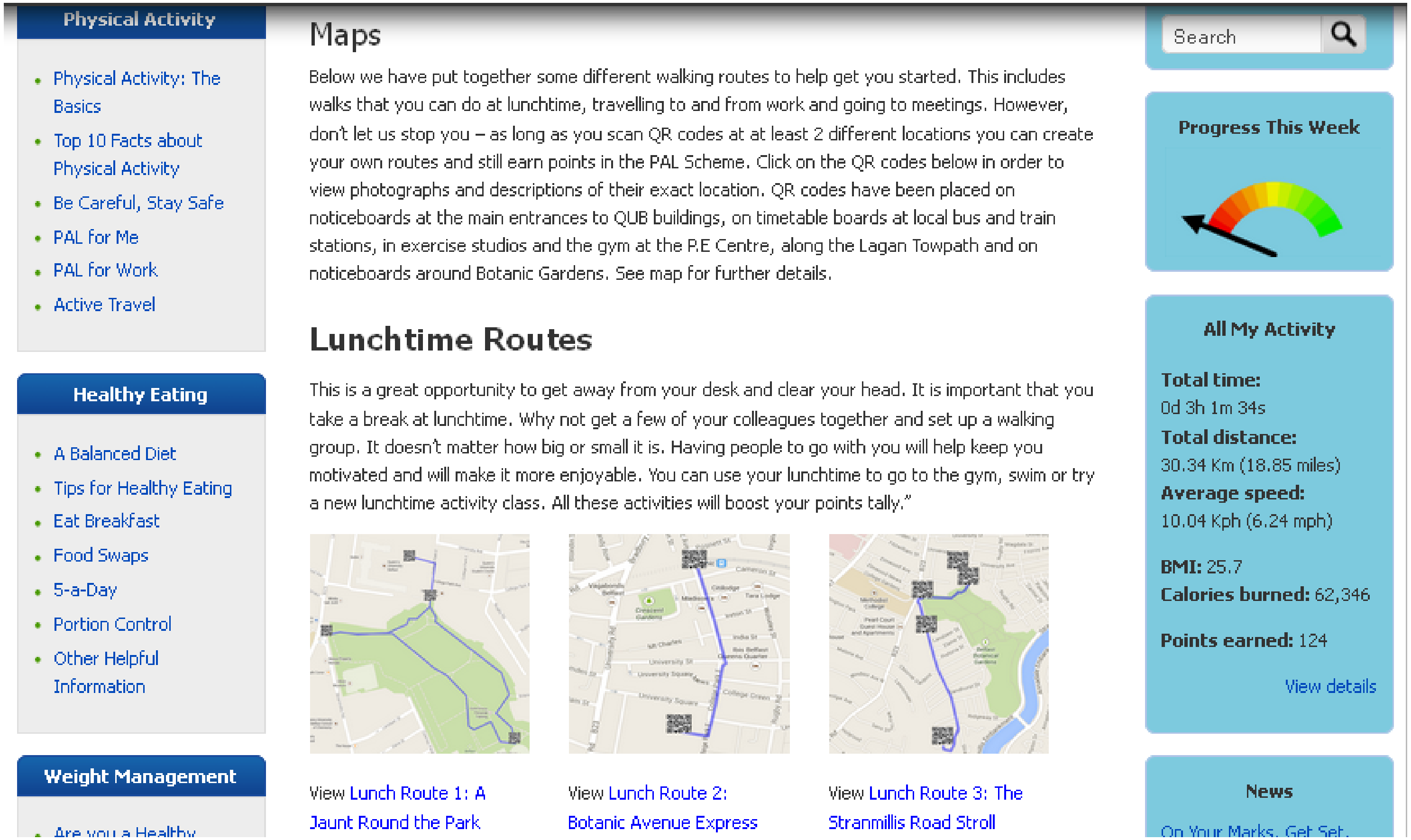

Participants can view maps highlighting various physical activity opportunities in the local environment, for example, suggested walks, local parks, gym, swim and exercise classes.

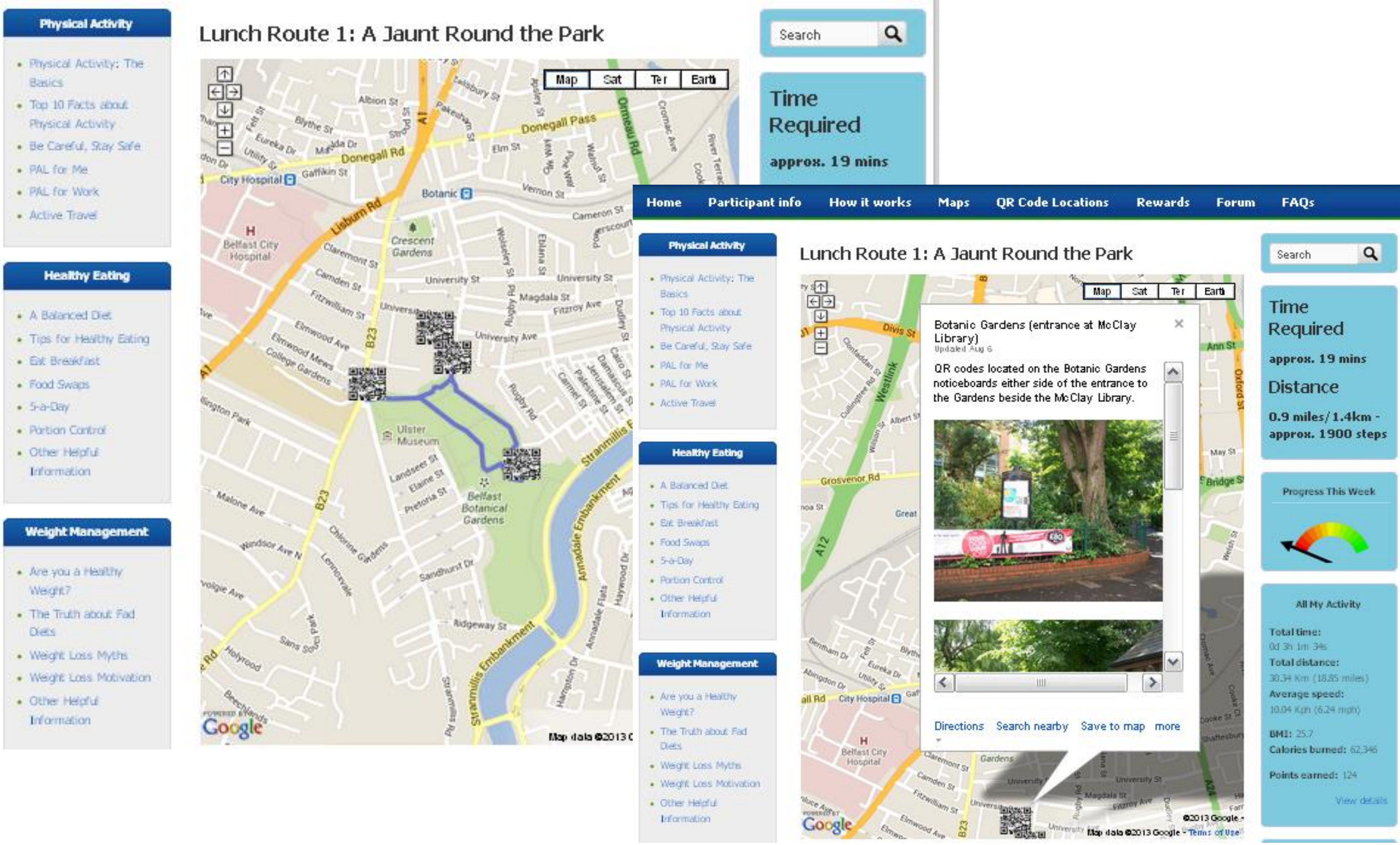

Participants can click on the map to find out more information about the walking route, including estimated time and distance. In this example, clicking on the QR code symbols enables participants to view a photograph and text description of the location of the sensors for monitoring and logging their physical activity session.

Additional functionality

- This is an open resource with new features and functionality continually being added to this platform

- Please feel free to suggest any additional features you would like added to the platform.

- For more information, please contact Dr Ruth Hunter; e-mail: ruth.hunter@qub.ac.uk; Tel: 02890978944

Contact details

If you wish to use the web platform or have any questions please get in touch

Dr Ruth Hunter
NIHR Career Development Fellow
Lecturer in Physical Activity and Public Health

UKCRC Centre of Excellence for Public Health Research
Queen's University Belfast
Northern Ireland, UK

E-mail: ruth.hunter@qub.ac.uk
Tel: 02890978944
Twitter: @ Ruth_HunterQUB
